# On the Management of Patients with Typhoid Fever

**Published:** 1898-10

**Authors:** S. Sols Cohen


### ON THE MANAGEMENT OF PATIENTS WITH TYPHOID FEVER.

Taking for granted that the well-known rules for diet are observed (and as to diet I advise small quantities of the most easily absorbable food—preferably pancreatized milk or expressed beef juice, administered every two or three hours) and that when the hygiene of the sick room is properly cared for, water, and often cold water, is the one agent of greatest usefulness in the management of patients suffering from enteric fever. It should be used freely in every case, internally as well as externally. Too often, nurses, unless instructed, will wait for the patient to ask before offering him water to drink. Sometimes the patient is too dull to realize even the sensation of thirst. Nurses should be instructed to give at least a quart of water in every twenty-four hours; boiled water, if there be any doubt of its purity. In many cases systematic sponging with cool or cold water will fulfill all the indications for external hydrotherapy. The sponging must be thoroughly and properly done. Nurses must be specifically and carefully instructed in its details. In cases severe from the outset, or which become severe in spite of treatment, the systematic cold bath should be instituted. The inexpert will do better by following the rigorous method of Brand, than by attempting to modify the method. The experienced will introduce such modifications as each individual case seems to require. Many nurses fail to prepare the bed properly for the reception of patients after the cold bath, and it is necessary for the physician to give specific instructions to have heated blankets ready, to receive the patient on a warm sheet which is to be tucked in, so as to prevent two wetted skin surfaces from coming in contact, and which can be used for drying the patient, and is then to be removed so as to permit him to lie between the two warm blankets. The use of red wine rather than whisky to give the patient before and after the bath is advisable. In some cases the use of aromatic spirit of ammonia answers the purpose. Between the tenth and twelfth days, it is doubtful whether plunging should be begun. After the twelfth day, the inexpert should never begin plunging. Plunging begun earlier should be continued or discontinued, according to circumstances. When plunging is not well borne, as for any reason it has not been instituted, frequent cold or cool sponging should be carried out. This is partly to reduce temperature but largely, like the bathing, to promote general metabolism, to stimulate excretion, and to keep up the tone of the peripheral vessels. The effects on temperature, pulse, respiration, excretions, sleep and general comfort must be the guides as to time, temperature and other details of the external application of water, whether as plunging or sponging. Too great a fall of temperature after sponge bath or plunge bath is harmful. A fall of, at most, one degree centigrade is enough for a single bath. Nor should patients be waked every two or three hours to have temperature taken or to be sponged or bathed. They should be allowed to sleep undisturbed for four or five hours, even when the applications are being made every second hour during wakefulness.

To reduce temperature, should this be thought necessary, and to prevent or control tympanites or hemorrhage, the continuous application of ice to the abdomen—usually over the right iliac fossa—is useful. Sometimes it is advisable to intermit the use of ice or to alternate the application of ice to the head and abdomen. In cases of severe nervous and cerebral symptoms, or very high temperature, there may be continuous application of ice to both head and abdomen. McCormick has had excellent success with the use of guaiacol externally.

Judicious internal medication is useful. The bowels should be cleansed by enema on admission (unless after the tenth day), after which, according to circumstances, a few small doses or one large dose of mercurous chloride (calomel) should be given. After the "calomel stool," intestinal disinfectants should be employed. These may not kill Eberth's bacillus, or neutralize its toxins, or chase after it into the spleen or cerebrum but they do render the patient's intestine a less favorable breeding ground for this organism and its many named and unnamed congeners; they do diminish the formation and hence the absorption of various named and unnamed toxins; they do render the course of the case less severe. Of drugs of this class, one may use guaiacol or its combinations, of which I prefer the carbonate; phenyl salicylate (salol); betanaphthol, or its benzoyl compound (benzonaphthol); creosote, or its carbonate (creosotal); carbolic acid and iodine, and the like. I usually employ salol or guaiacol carbonate, in doses of about five grains every second to fourth hour; more recently I have used benzonaphthol in doses of ten to fifteen grains. When diarrhœa is troublesome, bismuth salicylate may be used with the more powerful antiseptic. Betanaphthol-bismuth has been highly recommended. Should constipation be a feature of the case, an enema may be used every forty-eight hours, except during the period when ulceration is at its height, say from the twelfth to the sixteenth day, when the bowels should be let alone.

If, notwithstanding the free use of water, the urine is not excreted in sufficient quantity (that is, if it be less than thirty ounces in a day) some mild diuretic, as solution of ammonium acetate, or sweet spirit of nitre, or infusion of buchu, should be given. This is rarely necessary as the water drunk is usually an efficient diuretic, and the stimulation of the skin likewise assists excretion.

When the tongue is dry, harsh, fissured, covered with brownish fur, turpentine is useful beyond doubt. Sufficient must be given; about fifteen drops in emulsion or syrup of acacia every second or fourth hour. Should any sign of renal irritation develop, turpentine must be abandoned. I have, however, never seen it do harm, and have seen it do good too often to be laughed out of its use. It also served well in cases of tympanites or hemorrhage. To help restrain hemorrhage, calcium chloride is sometimes useful.

During the second week strychnine is useful in small doses, say about 1-100 to 1-60 grain every second to sixth hour. During the third week it may be increased to 1-30 grain every third hour, if need be. Alcohol is rarely necessary before the third week and often unnecessary throughout.

The principal aims of treatment are to give rest, physical and mental; to regulate rather than to interfere with the development of the course of affairs toward recovery; to avert danger of hemorrhage by precautions in diet; to avoid overburdening the digestion, yet to nourish adequately; to keep the bowel clean and reduce sepsis; to maintain the secretions; to keep up the peripheral circulation; to do no useless drugging.—DR. S. SOLS COHEN, in the *Pennsylvania Medical Journal.*

